# CCR2 and CXCR4 regulate peripheral blood monocyte pharmacodynamics and link to efficacy in experimental autoimmune encephalomyelitis

**DOI:** 10.1186/1476-9255-6-32

**Published:** 2009-11-11

**Authors:** Yuanfan Wang, Long Cui, Waldemar Gonsiorek, Soo-Hong Min, Gopinadhan Anilkumar, Stuart Rosenblum, Joseph Kozlowski, Daniel Lundell, Jay S Fine, Ethan P Grant

**Affiliations:** 1 Schering-Plough Research Institute, 2015 Galloping Hill Road, Kenilworth, NJ 07033, USA; 2 Hoffmann-La Roche, Inc., 340 Kingsland St., Nutley, NJ 07110, USA

## Abstract

**Background:**

CCR2 plays a key role in regulating monocyte trafficking to sites of inflammation and therefore has been the focus of much interest as a target for inflammatory disease.

**Methods:**

Here we examined the effects of CCR2 blockade with a potent small molecule antagonist to determine the pharmacodynamic consequences on the peripheral blood monocyte compartment in the context of acute and chronic inflammatory processes.

**Results:**

We demonstrate that CCR2 antagonism *in vivo *led to a rapid decrease in the number of circulating Ly6C^hi ^monocytes and that this decrease was largely due to the CXCR4-dependent sequestration of these cells in the bone marrow, providing pharmacological evidence for a mechanism by which monocyte dynamics are regulated *in vivo*. CCR2 antagonism led to an accumulation of circulating CCL2 and CCL7 levels in the blood, indicating a role for CCR2 in regulating the levels of its ligands under homeostatic conditions. Finally, we show that the pharmacodynamic changes due to CCR2 antagonism were apparent after chronic dosing in mouse experimental autoimmune encephalomyelitis, a model in which CCR2 blockade demonstrated a dramatic reduction in disease severity, manifest in a reduced accumulation of monocytes and other cells in the CNS.

**Conclusion:**

CCR2 antagonism *in vivo *has tractable pharmacodynamic effects that can be used to align target engagement with biologic effects on disease activity.

## Background

CCR2 plays a central role in two monocyte migration pathways. First, the constitutive recruitment of newly-formed monocytes that express high levels of the marker Ly6C (Ly6C^hi^) from the bone marrow to the peripheral blood is directed by the constitutive production of the CCR2 ligands monocyte chemotactic protein-1 (CCL2) and monocyte chemotactic protein-3 (CCL7) [1,2]. Second, the induced expression of CCR2 ligands at sites of tissue injury and inflammation can lead to the recruitment of monocytes directly to those tissues, where activation and differentiation into macrophage populations occurs [3]. As a source of TNFα, IL-1β, IL-6 and other pro-inflammatory mediators, macrophages are considered a major contributor to chronic inflammatory processes underlying diseases such as rheumatoid arthritis, multiple sclerosis, and asthma [4,5]. CCR2 deficient (CCR2^-/-^) mice exhibit reduced monocyte egress from the bone marrow and trafficking to sites of experimentally-induced inflammation, predicting that CCR2 blockade may be a viable inflammatory disease target [1,2,6]. Consequently, much interest has been placed on developing biologic and small molecule antagonists of CCR2 - mediated monocyte migration.

Very limited information has been published that describes the effects of small molecule CCR2 antagonists *in vivo*. In one report, INCB3344 was shown to be a potent CCR2 antagonist, blocking binding of and migration toward the ligand CCL2 *in vitro *[7]. This compound demonstrated activity in a variety of *in vivo *inflammatory models, such as thioglycollate-induced macrophage recruitment to the peritoneal cavity, DTH responses, and mouse experimental autoimmune encephalomyelitis (EAE). However, no pharmacodynamic assay was described in this report to provide a link between the activity of the compound *in vitro *and the efficacy observed *in vivo *and to provide a demonstration of target engagement *in vivo*. Further, the potency of INCB3344 in chemotaxis assays performed *in vitro*, reflected in low IC_50 _values, may underestimate the EC_50 _of the compound *in vivo*, where high levels of protein binding often substantially reduce the potency of small molecule antagonists. Here we characterized a potent CCR2 antagonist *in vitro*, accounting for any reduction in potency due to protein binding, and defined its pharmacodynamic effects on monocyte homeostasis and migratory potential *in vivo*. Further, we used pharmacodynamic measures to demonstrate that the efficacy of MK0812 in mouse EAE was linked to its pharmacodynamic modulation of CCR2 activity.

CXCR4 plays an important role in the retention of B cell precursors and neutrophils in the bone marrow [8]. CXCR4 deficiency and small molecule CXCR4 antagonists such as AMD3100 lead to a redistribution of neutrophils from the bone marrow to the periphery [8,9]. However, to date it has not been appreciated that CXCR4 regulates monocyte trafficking or retention in the bone marrow. In the course of these studies, we demonstrated that CXCR4 plays a role regulating monocyte redistribution to the bone marrow when CCR2 function is blocked. These data provide pharmacological evidence for a homeostatic balance between CXCR4-mediated retention of cells in the bone marrow and CCR2-mediated egress of monocyte into the peripheral blood.

## Methods

### Mice and reagents

BALB/cJ and C57BL/6J mice were obtained at 6-8 weeks of age and housed under specific pathogen free conditions. CCR2^-/- ^mice were obtained from Deltagen, Inc. The mice were maintained on a mixed C57BL/6 × 129Sv background. Homozygous wildtype CCR2^+/+ ^and CCR2^-/- ^littermates were obtained from heterozygous mating pairs. All animal studies were performed in compliance with the Institutional Animal Care and Use Committee of the Schering-Plough Research Institute. The CCR2 antagonist MK0812 was synthesized according to the method described in Patent US6812234 [10].

### CCR2 membrane binding assay

The radio-ligand binding assay was done using scintillation proximity assay (SPA) technology as previously described with some modifications [11]. Membranes (6 μg per assay point) from Ba/F3 cells transfected with mouse CCR2), and wheat germ agglutinin-coated SPA beads (450 μg per point; Amersham, Arlington Heights, IL), were pre-incubated for 30 min at room temperature in mouse plasma (C57BL/6 mouse, anticoagulant - sodium citrate; Bioreclamation, Inc., Hicksville, NY) containing 10 mM HEPES, pH 7.2. Then, prebound to WGA-SPA beads membranes were incubated at room temperature for 6 h with the indicated concentrations of CCR2 antagonists (in mouse plasma, 1% DMSO, final) and 0.2 nM ^125^I-rhCCL2 in mouse plasma (S.A. 2200 Ci/mmol, PerkinElmer Life and Analytical Science, Boston, MA). Binding was measured using a 1450 Microbeta Trilux counter (Wallac, Gaithersburg, MD). Binding constants (IC_50 _and slope) were calculated using GraphPad Prism software (GraphPad Software, Inc., La Jolla CA).

### CCR2 chemotaxis assay

Chemotaxis experiments were performed as described previously with some modifications [11]. WeHi-274.1 cells (ATCC) were resuspended at 4 × 10^6 ^cells/ml in mouse plasma (C57BL/6 mouse, anticoagulant - sodium citrate; Bioreclamation, Inc.) containing 10 mM HEPES, pH 7.2. Various concentrations of rmCCL2, diluted in mouse plasma, were dispensed at 30 μl/well into disposable microchemotaxis plates (ChemoTx 101-5sp; Neuro Probe, Gaithersburg, MD), which were then covered with a filter. Cells were pre-incubated at 37°C with various concentrations of MK0812 in a CO_2 _incubator for 30 min, and then 25-μl cell aliquots were applied to each spot on the filter. After incubation for 2 h at 37°C in a 5% CO_2 _incubator, the filters were removed. Migrated cells in the bottom wells were transferred to a Microlite luminometer plate (Thermo Fisher Scientific, Waltham, MA), and assayed using CellTiter-Glo kit according to the manufacturer's instructions (Promega, Madison, WI). The area under the curve (AUC) and inhibition constants (IC_50 _and slope) were calculated using GraphPad Prism software.

### Chemokine-induced monocyte migration in vivo

Recombinant mouse CCL2 and CCL3 (R & D Systems, Minneapolis, MN) were dissolved in PBS and injected i.v. in the lateral tail vein of BALB/c mice, CCR2^-/- ^mice or CCR2^+/+ ^littermate controls. 30 min later, chemokine-injected and control animals were euthanized, blood was collected into EDTA-coated tubes. Plasma was collected after centrifugation and frozen at -20°C until used for measurements of CCL2, CCL7 or CXCL12 by ELISA (R & D Systems). Where indicated, animals were dosed with the CXCR4 antagonist AMD3100 (Sigma-Aldrich, St. Louis, MO) subcutaneously 1 h prior to dosing with MK0812 by oral gavage (p.o.). In all studies involving MK0812, the compound was dosed as a solution in 0.4% hydroxypropyl methylcellulose (MC) via oral gavage 1 h prior to CCL2 or CCL3 administration.

### Blood cell differential counts and flow cytometry

White blood cell differential counts to quantify total leukocytes, lymphocytes, monocytes, neutrophils, basophils and eosinophils were performed using mouse whole blood collected into EDTA-coated tubes. Blood samples were analyzed using the ADVIA 120 Hematology System (Bayer HealthCare, LLC, Tarrytown, New York) according to the manufacturer's instructions for analysis of mouse blood.

To measure the number of CD11b^+^Ly6C^hi ^monocytes, blood was collected from euthanized mice into EDTA-coated tubes. Blood was aliquoted into 96-well V-bottom plates. To block nonspecific binding, normal rat serum was added to a final concentration of 20% and incubated for 10 min at room temperature. Blood was then incubated with PerCP-Cy5.5-anti-CD11b, PE-antiLy6G, FITC-anti-Ly6C and/or anti-CXCR4 antibodies or isotype controls (BD Biosciences, Franklin Lakes, NJ) for 20 min at RT. The cells were pelleted by centrifugation and erythrocytes were lysed using FACS Lysing Solution (BD Biosciences) according to the manufacturer's instructions. Cells were washed and analyzed on a FACS Calibur instrument (BD Biosciences).

### Experimental autoimmune encephalomyelitis

EAE was induced in C57BL/6 female mice at 10-12 weeks of age. Mice were injected with 200 ng pertussis toxin (Sigma-Aldrich, St. Louis, MO) i.v. on days 0 and 2. On day 1, mice were immunized with 50 μg MOG_35-55 _peptide (Princeton Biomolecules, Lanhorne, PA) emulsified with 300 μg heat-killed Mycobacterium tuberculosis (Difco, Detroit, MI) and injected in two 50 μl s.c. injections over the flanks. Beginning on day 5 post immunization, animals were dosed with FTY720 (1 mg/kg, q.d.) or MK0812 (10 or 30 mg/kg, b.i.d.) by oral gavage. Both compounds were dissolved in 0.4% hydroxypropyl methylcellulose (MC). Control animals were dosed b.i.d with MC. Animals were scored for clinical symptoms daily using the following scoring system: 0, no signs of disease; 1, tail paralysis; 2, tail paralysis and hind limb weakness; 3, hind limb and tail paralysis; 4, hindlimb and forelimb paralysis; 5, moribund or dead. Mice that reached a score of 4 that did not improve within 24 h were euthanized and assigned a score of 5 for humane reasons.

### Analysis of central nervous system (CNS) infiltrates

To assess the infiltration of leukocytes into CNS tissues in the EAE model, naïve mice or mice with EAE that had been dosed with either MC (the vehicle) or MK0812 (30 mg/kg, b.i.d.) were euthanized and then perfused with 50 ml cold PBS containing 2% glucose and heparin (2.1 U/ml). The spinal cord and brains were dissected and mononuclear cells isolated using a Neural Tissue Dissociation Kit (Papain) (Miltenyi Biotec, Inc., Auburn, CA) according to the manufacturer's instructions. Isolated mononuclear cells were then stained for flow cytometric analysis using fluorochrome-conjugated anti-CD45, anti-CD11b, anti-CD3, anti-Ly6G, anti-Ly6C antibodies (BD Biosciences), washed and analyzed on a FACS Calibur instrument (BD Biosciences).

### Statistical analysis

Statistical comparisons between groups were performed using Kruskal-Wallis ANOVA analyses using GraphPad Prism software. P values < 0.05 were considered statistically significant.

## Results

### CCL2 induces monocyte migration *in vivo*

**Figure 1:**
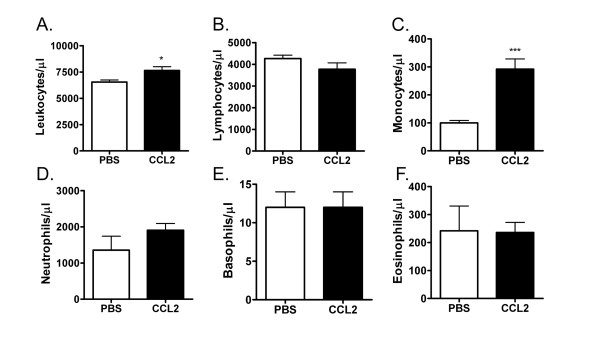
**Administration of CCL2 *in vivo *leads to a selective increase in circulating monocytes**. BALB/c mice were injected with 300 ng CCL2 or PBS i.v. into the tail vein. 30 min later, blood was collected and analyzed for white blood cell counts using an ADVIA system to quantify (A) total leukocytes, (B) lymphocytes, (C) monocytes, (D) neutrophils, (E) basophils and (F) eosinophils. ***p < 0.001 compared to PBS group. The data presented are the mean ± S.E.M. of six mice per group and are representative of three independent experiments.

**Figure 2:**
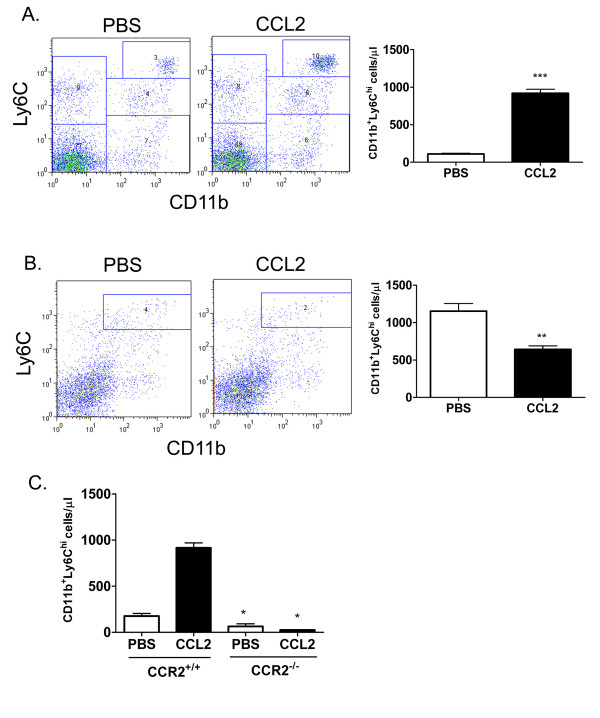
**Administration of CCL2 *in vivo *leads to a CCR2-dependent increase in peripheral blood Ly6C^hi ^monocytes**. BALB/c mice were injected with 300 ng CCL2 or PBS i.v. into the tail vein. 30 min later, blood (A) and bone marrow (B) were collected and stained for expression of CD11b and Ly6C. Neutrophils were excluded by gating on the Ly6G-negative population. ***p < 0.001 and **p < 0.01 compared to PBS group. C, CD11b^+^Ly6C^hi ^monocyte numbers were determined in the blood of CCR2^+/+ ^and CCR2^-/- ^mice 30 min after PBS or CCL2 injection. *p < 0.05 PBS WT versus PBS KO group and CCL2 WT versus CCL2 KO group. The data presented are the mean ± S.E.M. of six mice per group and are representative of three independent experiments.

CCR2 plays an important role in regulating the homeostatic recruitment of newly-formed monocytes from the bone marrow to the peripheral blood [1,2]. To begin to examine the mechanism behind this process, we first assessed whether intravenous administration of a bolus of CCL2 would lead to detectable changes in the circulating pool of monocytes and other major blood leukocyte cell populations. To this end, white blood cell differential counts were performed to measure various cell types in mouse whole blood 30 min after administration of CCL2 relative to control mouse blood. Under these conditions, a three-fold increase in peripheral blood monocyte numbers was detected, with no significant change in the other major cell populations, including neutrophils, lymphocytes, basophils or eosinophils (Figure 1). This selective effect on the monocyte population prompted us to measure changes in the circulating monocyte pool with a more specific methodology, whereby newly formed monocytes can be detected by flow cytometry based on expression of the Ly6C marker. We determined that a bolus injection of CCL2 led to a five-fold increase in the number of blood monocytes, as defined by lack of expression of the neutrophil marker Ly6G and expression of CD11b and Ly6C (CD11b^+^Ly6C^hi^) (Figure 2A). A corresponding reduction in cell number of the corresponding population of cells in the bone marrow was detected in the same animals, consistent with an increased recruitment of monocytes from the bone marrow to the peripheral blood (Figure 2B). This effect was clearly mediated through CCR2, since no such increase in peripheral blood monocyte population was observed when CCL2 was administered to CCR2^-/- ^animals (Figure 2C).

### The CCR2 antagonist MK0812 blocks CCR2 function *in vitro*

**Figure 3:**
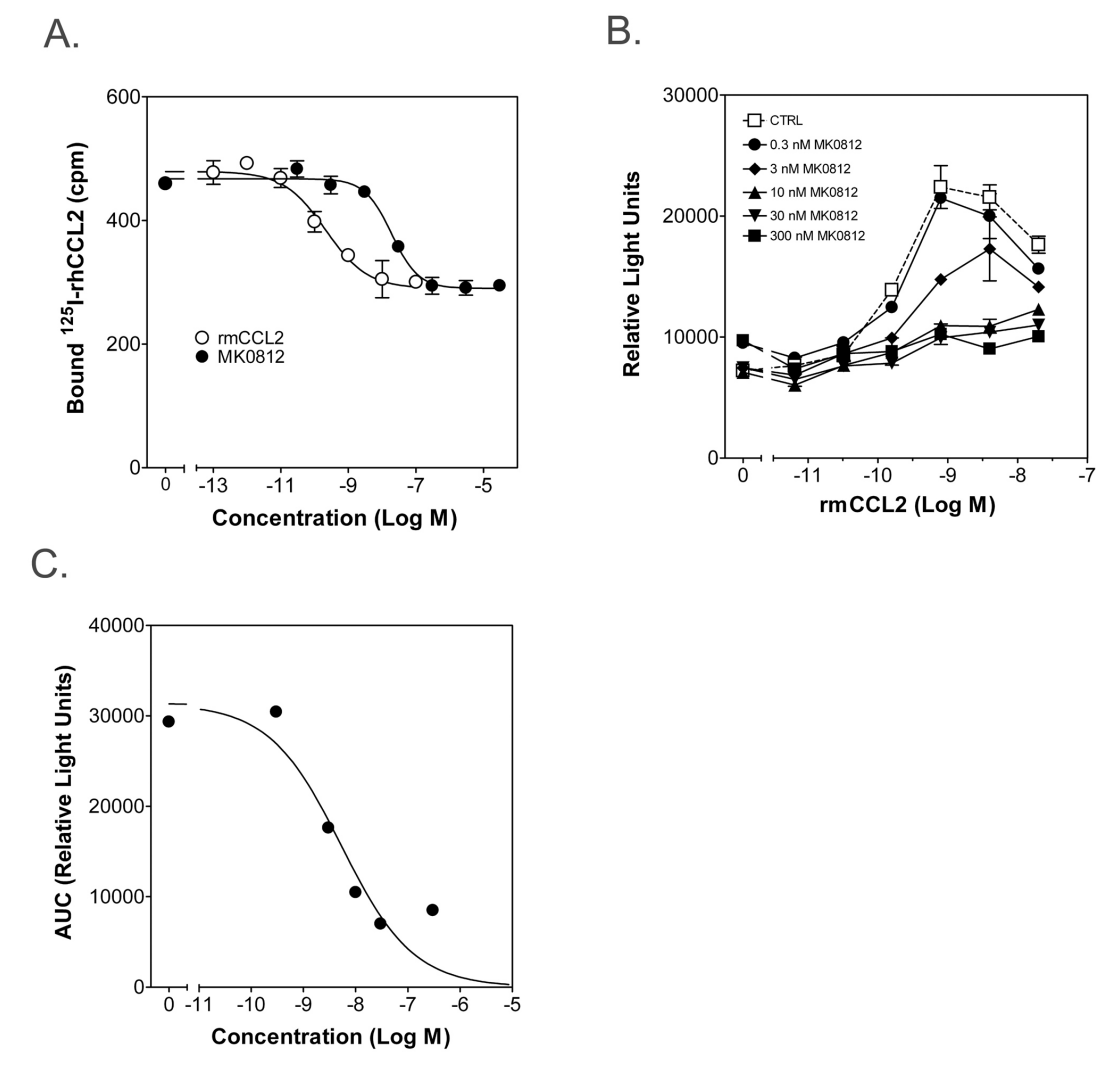
**MK0812 inhibits binding to CCR2 and CCL2-mediated chemotaxis *in vitro***. A, Inhibition of binding of ^125^I-rhCCL2 to membranes from Ba/F3 cells transfected with mouse CCR2 was tested using the indicated concentrations of MK0812 or unlabeled rmCCL2. B, The inhibition of WeHi-274.1 cell chemotaxis was tested with the indicated concentrations of MK0812. C, The area under the curves from the data depicted in (B) were calculated and plotted against the concentration of CCL2 to determine an IC_50 _= 5 nM for MK0812. The data presented are the mean ± S.E.M. and are representative of three independent experiments.

Having determined that this CCL2-induced monocyte migration *in vivo *was CCR2 mediated, we used this system to characterize the effects of the small molecule CCR2 antagonist MK0812, a highly potent and selective reagent [12,13]. We first confirmed the potency of this compound against CCR2 using a membrane binding assay. MK0812 inhibited the binding of ^125^I-labeled recombinant human CCL2 to CCR2-containing mouse cell membranes with an IC_50 _of 19 nM in the presence of 97% mouse plasma, accounting for any shift in potency due to protein content *in vivo *(Figure 3A). Similarly, MK0812 potently inhibited CCL2-mediated chemotaxis of WEHI-274.1 cells with an IC_50 _= 5 nM in the presence of 99% mouse plasma (Figure 3B, C). Having defined the high potency of this molecule against mouse CCR2, we sought to characterize its effects on monocyte migration *in vivo*.

### Pharmacokinetic analysis of MK0812

We next evaluated the pharmacokinetic behavior of MK0812 *in vivo*. Mice were dosed with MK0812 at 50 mg/kg, p.o. and plasma was collected 1, 4, 8, or 16 h later to determine the plasma exposure over time. The compound displayed a T_1/2 _= 1.6 h, with C_max _= 27 μM and an AUC = 104 μM·h (data not shown). Thus, 16 h after a 50 mg/kg dose, the plasma concentration of MK0812 was 50 nM, or ten-fold higher than the IC_50 _for inhibition of chemotaxis in the presence of 99% mouse plasma *in vitro*. Substantially lower doses would therefore be required for complete inhibition of CCR2 activity in acute *in vivo *assays of 2-3 h duration, as described below.

### Pharmacodynamic effects of the CCR2 antagonist MK0812

**Figure 4:**
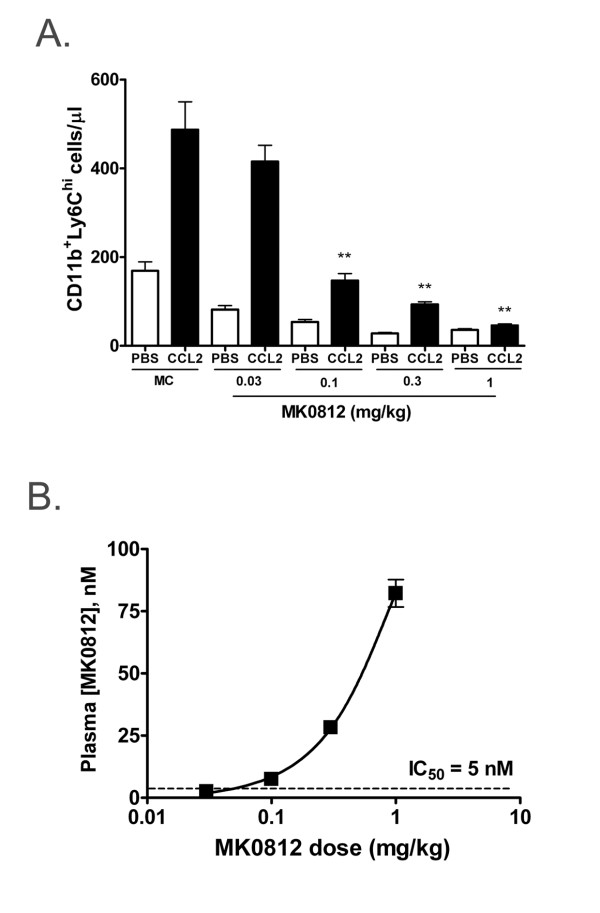
**MK0812 inhibits CCL2-mediated monocyte migration *in vivo***. A, BALB/c mice were administered the indicated dosage of vehicle (MC) or MK0812 by oral gavage. 1 h later, the mice were injected with PBS or 300 ng CCL2 i.v. 30 min later, peripheral blood CD11b^+^Ly6C^hi ^monocyte numbers were determined as in Figure 2. B, The plasma concentrations of MK0812 were determined from the animals used in (A) and plotted versus the dosage of MK0812 administered. The IC_50 _for inhibition of CCL-2 mediated chemotaxis (see Figure 3) is indicated by a dashed line. The data presented are the mean ± S.E.M. of six mice per group and are representative of three independent experiments. **p < 0.01 compared to MC/CCL2 group.

We next sought to assess the effect of MK0812 on CCR2-dependent monocyte migration *in vivo*. As shown above, intravenous injection of a 300 ng bolus of CCL2 led to a clear increase in the number of blood CD11b^+^Ly6C^hi ^monocytes (Figure 4A). Oral administration of MK0812 1 h prior to the CCL2 challenge dose-dependently and completely inhibited this response. We determined the concentration of MK0812 present in the plasma at the time of blood collection in order to define the pharmacokinetic - pharmacodynamic relationship for this compound. We observed a linear relationship between the administered dose of MK0812 and the levels of the compound present in the plasma. The most complete inhibition of the CCL2-driven increase in monocyte number was observed at doses that achieved plasma concentrations of MK0812 above the *in vitro *IC_50 _for this compound (Figure 4B). Thus, the *in vivo *effects of MK0812 align well with the predicted potency based on the *in vitro *characterization of the compound.

**Figure 5:**
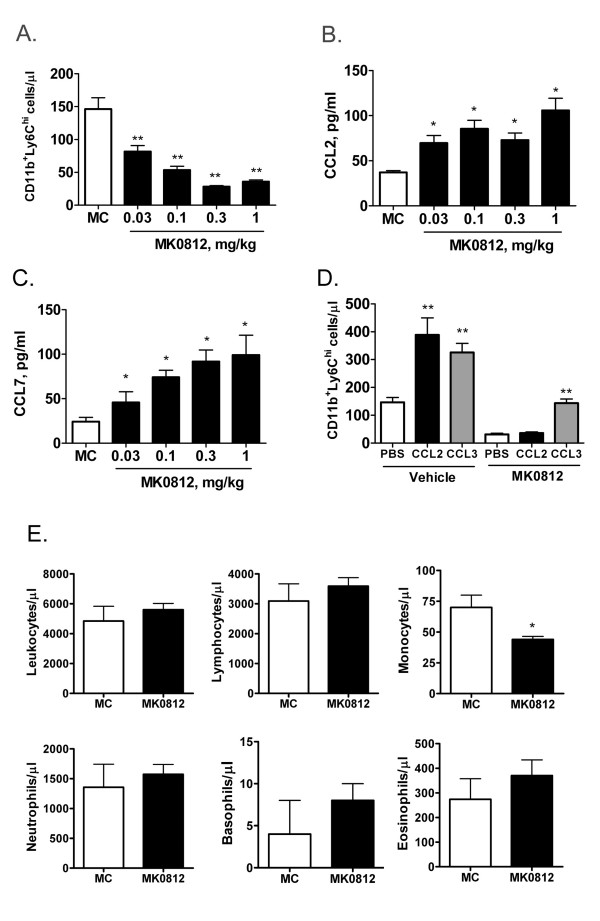
**Pharmacodynamic effects of MK0812 *in vivo***. BALB/c mice were administered the indicated dosage of vehicle (MC) or MK0812 by oral gavage. 2 h later, peripheral blood CD11b^+^Ly6C^hi ^monocyte numbers were determined as in Figure 2 (A) and plasma concentrations of CCL2 (B) and CCL7 (C) were determined. ***p < 0.001 and **p < 0.01 compared to MC group. D, The effect of MK0812 (3 mg/kg, p.o.) or vehicle (MC) on CCL2 and CCL3 mediated changes in CD11b^+^Ly6C^hi ^monocyte numbers were evaluated as in Figure 4. **p < 0.01 compared to PBS group. E, BALB/c mice were dosed with vehicle (MC) or MK0812 (30 mg/kg, p.o.) and 2 h later, blood was collected and analyzed for white blood cell counts using an ADVIA system to quantify total leukocytes, lymphocytes, monocytes, neutrophils, basophils and eosinophils. *p < 0.05 compared to MC group. The data presented are the mean ± S.E.M. of six mice per group and are representative of three independent experiments.

In the course of this experiment, we noted that the circulating pool of CD11b^+^Ly6C^hi ^monocytes decreased in animals as a consequence of dosing with MK0812 (Figure 4A). This finding is likely a reflection of the role of CCR2 in the homeostatic process of monocyte recruitment from the bone marrow to the peripheral blood. In order to extend the characterization of the effects of CCR2 antagonist administration, we performed additional analyses of the blood from animals dosed with MK0812. As shown above, oral administration of MK0812 led to a dose-dependent reduction of up to 75% of circulating CD11b^+^Ly6C^hi ^monocytes (Figure 5A). In addition, the levels of CCL2 and CCL7, two important ligands for CCR2 implicated in recruitment from the bone marrow, were significantly elevated in mice dosed with MK0812 (Figure 5B, C). This effect was selective for CCR2 ligands, since no change was observed in plasma levels of a selection of other chemokines, including CCL3, CCL4, CXCL1, CXCL2, and CXCL12 (data not shown). Further, we repeated these studies using the structurally distinct CCR2 antagonist INCB3344 and observed similar pharmacodynamic effects on blood Ly6C^hi ^monocyte number and circulating CCL2 and CCL7 levels (data not shown). In addition, elevated levels of CCL2 and CCL7, but not other chemokines, was observed in the plasma of CCR2^-/- ^mice relative to CCR2^+/+ ^littermates, indicating that these changes in CCR2 ligand levels were general phenomena of CCR2 antagonism and absence of CCR2 expression (data not shown).

While CCR2 antagonism *in vivo *rendered CD11b^+^Ly6C^hi ^monocytes non-responsive to CCL2, we sought to determine if this monocyte population remained responsive to other chemotactic signals. We determined that intravenous administration of the CCR1 ligand macrophage inflammatory protein-1α (CCL3) into naive mice caused a recruitment of monocytes into the peripheral blood similar to that observed with CCL2 (Figure 5D). This result suggests that both CCR1 and CCR2 ligands can mediate the direct recruitment of monocytes from the bone marrow to the peripheral blood. Notably, while administration of MK0812 paralyzed the ability of this monocyte population to response to CCL2, responsiveness to CCL3 remained intact, such that CCL3 induced a 2.5-3-fold increase in Ly6C^hi ^monocyte number both in the presence and absence of MK0812 (Figure 5D). As discussed later, this finding may have important implications for the use of CCR2 antagonists to treat human disease. Finally, administration of MK0812 to mice caused a selective decrease in the circulating monocyte population, as white blood cell counts for the other major blood cell populations remained unchanged (Figure 5E).

### Role for CXCR4 in the modulation of peripheral monocyte number by MK0812

**Figure 6:**
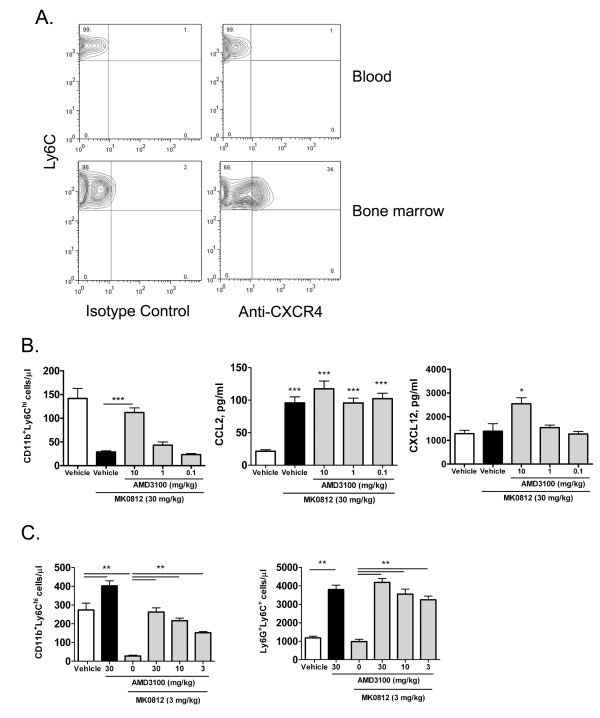
**The pharmacodynamic effect of MK0812 on monocytes is mediated by CXCR4**. A, Blood and bone marrow were isolated from naïve BALB/c mice and stained for expression of CD11b, Ly6G, Ly6C and CXCR4. Neutrophils were excluded by gating on the Ly6G^- ^population and monocytes were gated upon based on CD11b^+^Ly6C^hi ^expression. The expression of CXCR4 on the gated CD11b^+^Ly6C^hi ^monocytes is indicated. B, BALB/c mice were administered the indicated dosage of vehicle (MC) or AMD3100 s.c. 1 h later, mice were dosed p.o. with MK0812 or vehicle. 30 min later, peripheral blood CD11b^+^Ly6C^hi ^monocyte numbers were determined as in Figure 2. Plasma concentrations of CCL2 and CXCL12 were determined from the same mice. ***p < 0.001, *p < 0.05 compared to the Vehicle group. C, The effect of AMD3100 was assessed on Ly6G^-^CD11b^+^Ly6C^hi ^monocytes and Ly6G^+^Ly6C^+ ^neutrophils. **p < 0.01 as shown. The data presented are the mean ± S.E.M. of six mice per group and are representative of three independent experiments.

The reduction in circulating CD11b^+^Ly6C^hi ^monocytes that occurs after CCR2 antagonist treatment could be due to a recycling of monocytes from the blood back to the bone marrow or could simply reflect the further migration of monocytes into tissues where differentiation to macrophage and dendritic cell subsets occurs. One potential recycling mechanism could involve the stromal cell-derived factor-1 (CXCL12) - mediated retention of leukocytes mediated through CXCR4 [8,14]. We first examined the expression of CXCR4 on Ly6G^-^CD11b^+^Ly6C^hi ^monocytes in the blood and bone marrow of naïve mice. While CXCR4 was clearly detectable on 34% of bone marrow Ly6C^hi ^monocytes, we did not detect expression on the corresponding blood monocyte population (Figure 6A).

To assess the role of CXCR4 on the decrease in blood monocytes upon CCR2 antagonism, we treated animals with the selective CXCR4 antagonist AMD3100 prior to administration of MK0812. As shown above, in animals pretreated with vehicle alone, MK0812 caused a significant decrease in CD11b^+^Ly6C^hi ^blood monocytes (Figure 6B). Pretreatment with AMD3100 significantly and dose-dependently counteracted the effect of the CCR2 antagonist. These data suggest that the reduced peripheral monocyte number in CCR2 antagonist treatment is largely due to recycling of this population back into the bone marrow via CXCR4. In all animals dosed with the CCR2 antagonist, there was a clear elevation in plasma CCL2 levels, suggestive of comparable CCR2 receptor coverage in all groups (Figure 6B). An elevation in plasma CXCL12 levels was detected in animals treated with the highest dose of AMD3100, consistent with substantial coverage of CXCR4 at the 10 mg/kg dose (Figure 6B). Notably, this dose of AMD3100 also had the greatest impact on monocyte levels in the plasma.

To rule out the possibility that CXCR4 antagonism alone could cause an elevation in peripheral monocyte number independent of CCR2 antagonism, we examined whether mice dosed with AMD3100 alone affected peripheral monocyte and neutrophil populations. At the highest dose tolerable to mice, 30 mg/kg, AMD3100 had only a modest effect on the peripheral Ly6C^hi ^monocyte number relative to vehicle treated animals, while a clear reversal of the effects of MK0812 were again observed (Figure 6C). As expected, CXCR4 antagonism alone did lead to a substantial increase in peripheral blood Ly6G^+^Ly6C^+ ^neutrophils, an effect that was observed to be independent of CCR2 antagonism (Figure 6C). Thus, CXCR4-mediated retention of neutrophils in the bone marrow is a mechanism to limit neutrophil egress from the bone marrow under homeostatic conditions.

### The CCR2 antagonist MK0812 blocks EAE

**Figure 7:**
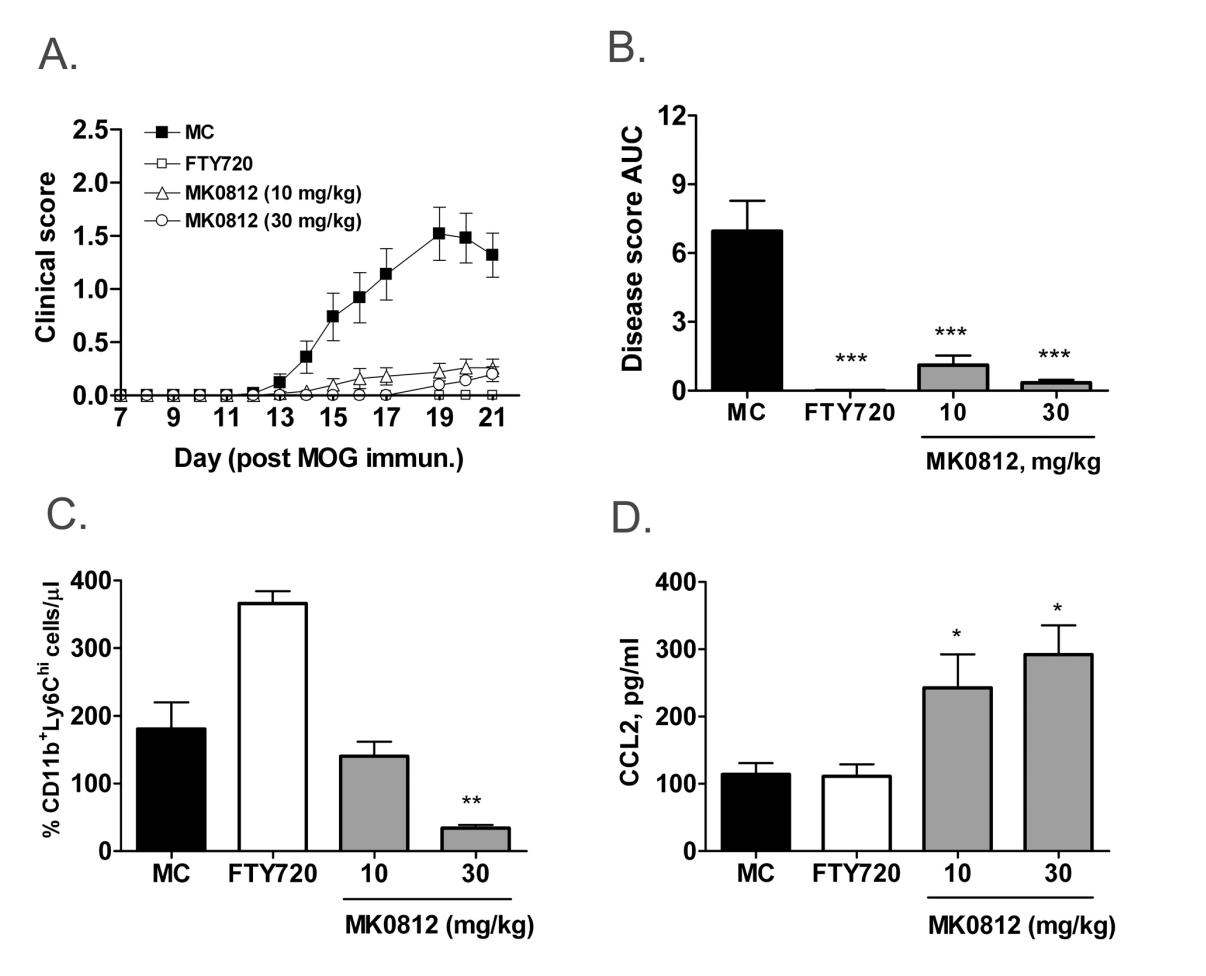
**Chronic MK0812 treatment inhibits EAE accompanied by pharmacodynamic effects**. A, Effect of MK0812 (10 mg/kg, b.i.d., p.o.) and FTY720 (1 mg/kg. q.d., p.o.) on EAE severity in C57BL/6J mice. Drug dosing was initiated at d5 after immunization with MOG_35-55 _peptide in CFA. B, The areas under the curve from the data depicted in (A) were calculated. C, Blood CD11b^+^Ly6C^hi ^monocyte numbers (C) and the plasma concentration of CCL2 (D) were determined from the mice at d21. ***p < 0.001, **p < 0.01, *p < 0.05 compared to MC group. The data presented are the mean ± S.E.M. of twnenty-five mice per group and are representative of three independent experiments.

The results presented above describe the acute effects of CCR2 antagonism *in vivo*. We sought to determine whether these findings could be extended to situations of chronic CCR2 antagonism in the context of disease modification. We therefore tested the effect of MK0812 in mouse experimental autoimmune encephalomyelitis (EAE), a model in which CCR2^+^Ly6C^hi ^monocytes play a crucial role in disease pathology [15]. C57BL/6 mice were immunized with myelin oligodendrocyte glycoprotein peptide residues 35-55 (MOG_35-55_). Five days after immunization, before clinical symptoms were evident, animals were divided into groups and dosed with either vehicle or MK0812 (10 or 30 mg/kg, b.i.d., p.o.). As a positive control, one group of animals was dosed with the sphingosine-1 phosphate receptor agonist FTY720 (1 mg/kg, q.d., p.o.), a therapeutic that has been shown to be efficacious in EAE models [16]. Clinical scores were assigned each day using the scoring system described in the *Methods *until day 21, when the study was terminated. As expected, FTY720 significantly and completely inhibited disease progression (Figure 7A, B). Strikingly, both doses of MK0812 almost completely blocked disease progression. There was moderate dose dependence evident in the data expressed as mean daily clinical scores or as the area under the curve (AUC) of the disease scores over the course of the experiment (Figure 7A and 7B, respectively).

At the termination of the study, we assessed the number of CD11b^+^Ly6C^hi ^monocytes in the peripheral blood. Notably, there was a dose-dependent reduction in this monocyte population in animals treated with MK0812. In contrast, animals treated with FTY720 showed an elevated monocyte number that likely is due to a corresponding reduction in number of circulating lymphocytes in these animals. In addition, MK0812-treated animals had elevated levels of CCL2 in the plasma (Figure 7D). This effect was specific to the groups treated with a CCR2 antagonist as there was no change in plasma CCL2 levels in mice dosed with FTY720. Therefore, the pharmacodynamic effects of CCR2 antagonism observed after acute treatment extend to chronic dosing regimens and provide biomarkers of CCR2 antagonism in the context of the EAE disease model. As such, these biomarkers may be of utility to gauge target engagement in the context of human clinical trials with CCR2 antagonists.

**Figure 8:**
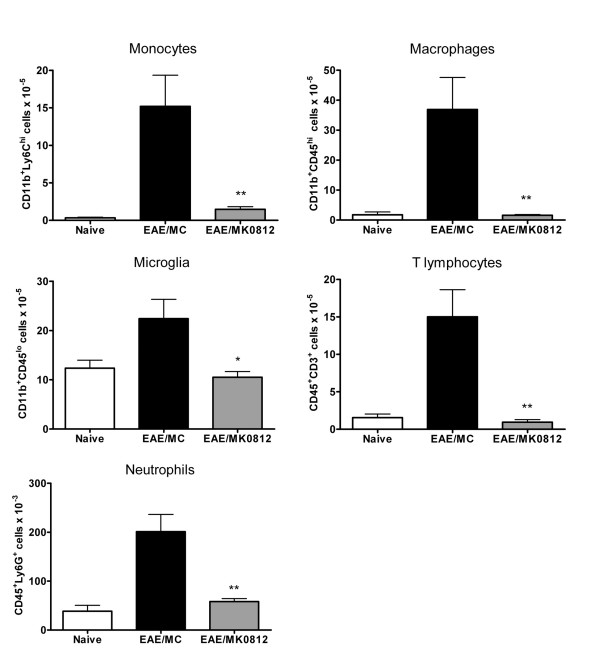
**Chronic MK0812 treatment inhibits the accumulation of monocytes, macrophages and other leukocyte populations in the CNS**. A, Leukocytes were isolated from naïve mice or mice immunized with MOG_35-55 _and dosed with MK0812 (30 mg/kg, b.i.d., p.o.) or MC as in Figure 7. Leukocyte isolations were performed between days 14-21, when clinical disease was evident in the EAE/MC group. The number of monocytes, macrophages, microglia, T lymphocytes and neutrophils were calculated based on flow cytometric analyses using the indicated combinations of cell surface markers. Cell numbers indicated are total cells per mouse. **p < 0.01 and *p < 0.05 compared to MC group. The data presented are the mean ± S.E.M. of five mice per group and are representative of two independent experiments.

The reduction in EAE disease severity as a result of MK0812 treatment may relate to the inhibition of recruitment of Ly6C^hi ^monocytes to the CNS, where activation and differentiation to the macrophage lineage could contribute to disease pathology. To assess the effect of MK0812 on leukocyte accumulation in the CNS, we isolated cells from the spinal cord and brain tissues of mice immunized with MOG_35-55 _and dosed with MC or MK0812 (30 mg/kg, b.i.d., p.o.) as described above. Flow cytometric analyses of the isolated cells indicated that elevations in CD11b^+^Ly6C^hi ^monocytes, CD11b^+^CD45^hi ^macrophages, CD11b^+^CD45^lo ^microglia, CD45^+^CD3^+ ^T lymphocytes, and CD45^+^Ly6G^+ ^neutrophils were evident in isolates from mice with EAE compared to naïve mice (Figure 8). Mice treated with MK0812 had significantly decreased levels of both Ly6C^hi ^monocytes as well as all other leukocyte populations that were analyzed. Therefore, the reduction in EAE clinical disease severity following MK0812 treatment is consistent with a reduced accumulation of CCR2-expressing Ly6C^hi ^monocytes and/or other cells.

## Discussion

For many years, it has been appreciated that monocytes are a highly dynamic population. In the mouse, over the span of 24 h, on the order of a million monocytes transit from the bone marrow to the peripheral blood, half of which migrate further into tissues, where differentiation to various macrophage and dendritic cell populations can occur [17]. Over the past several years, it has been appreciated that CCR2 plays an important role in regulating monocyte traffic from the bone marrow to the peripheral blood, providing a molecular basis for the continuous supply of newly formed monocytes. Here we demonstrated that blockade of CCR2 function with a small molecule antagonist leads to a rapid decline in the number of circulating newly formed CD11b^+^Ly6C^hi ^monocytes. These data demonstrate the highly dynamic nature of monocyte traffic under normal homeostatic conditions. Further, we show that CCL2 and CCL7 are present at detectable levels in normal mouse plasma. While the constitutive source for these chemokines has not been defined, it allows the formation of a gradient of the chemokine that directs monocyte traffic from the bone marrow to the blood. In the presence of a CCR2 antagonist *in vivo*, the levels of circulating CCL2 and CCL7 increased significantly, indicating that the continuous consumption of these factors, through interactions with CCR2, regulate their levels in the plasma. Similarly, the CXCR4 antagonist AMD3100 caused an increase in the basal levels of CXCL12 in the plasma, indicating a similar role for this receptor in clearance of its ligands. It appears to be a general phenomenon that chemokine receptor antagonism recapitulates the observation that CCR2, CXCR2, CXCR3 and CX3CR1 knockout mice each have elevated levels of their respective ligands [18].

The rapid decrease in monocyte number in the presence of a CCR2 antagonist prompted us to explore the mechanism behind this decrease. Since CXCL12-CXCR4 interactions are an important mechanism by which leukocyte populations are retained in the bone marrow, we examined whether a selective CXCR4 antagonist would impact the CCR2 antagonist effect on monocyte number. Our data indicate that blockade of CXCR4 greatly abrogated the decrease in monocyte number induced by a CCR2 antagonist, and is the primary if not only, explanation for this decrease. These data suggest the presence of a balance between CXCR4-mediated retention of monocytes in the marrow and CCR2-mediated export of these cells to the periphery. The balance between CXCL12 and CCL2 levels therefore appears to comprise a rheostat that regulates monocyte egress from the bone marrow. We also demonstrated that this balance can be shifted in favor of enhanced monocyte egress in that an i.v. injection of a bolus of CCL2 led to a rapid increase in CD11b^+^Ly6C^hi ^monocytes in the peripheral blood. Likewise, in the context of inflammation where CCR2 ligands are produced, a direct recruitment of monocytes from the bone marrow into the blood and eventually to inflamed tissues is conceivable. In these studies, we demonstrated the expression of CXCR4 on bone marrow Ly6C^hi ^monocytes, but did not detect expression of this receptor on blood monocytes. These data suggest that CXCR4 expression is highly dynamic and may be down-regulated rapidly around the time of egress from the bone marrow to peripheral blood. Since CXCR4 appears to play a role in blood monocyte trafficking when CCR2 is antagonized, CXCR4 expression may be highly dynamic in this monocyte population, such that CXCR4 expression is restored upon CCR2 antagonism.

Notably, paralysis of CCR2 with an antagonist did not affect the ability of monocytes to respond to CCR1-directed recruitment to the blood. This finding is consistent with *in vitro *chemotaxis data that indicate that selective chemokine receptor antagonism does not affect the ability of cells to migrate in response to other receptors present on the same cell. These data indicate that CCR2 blockade does not preclude the ability of monocytes to be recruited from the bone marrow via other receptors. One implication of this result is that CCR2 antagonists alone may be inadequate to block inflammatory conditions in which production of ligands for multiple monocyte chemokine receptors occurs. This redundancy has been appreciated for some time and may explain the lack of clinical efficacy of selective biologic and small molecule CCR2 antagonists in diseases such as rheumatoid arthritis [19,20]. On the other hand, some degree of redundancy may be viewed as sparing - complete blockade of all avenues of monocytes recruitment to tissues might be unacceptable in terms of host defense and immunosurveillance. Finding the right balance between these two issues remains a challenge for the development of chemokine receptor antagonists suitable for clinical use.

Finally, we demonstrate clear efficacy of a potent CCR2 antagonist in EAE. These data extend previous observations with another small molecule antagonist, INCB-3344, in this model and are consistent with the decreased susceptibility of CCR2^-/- ^mice to EAE [6,7]. Two pharmacodynamic markers of CCR2 antagonism, a decrease in peripheral blood CD11b^+^Ly6C^hi ^monocytes and an elevation in plasma CCL2, were evident after chronic dosing with MK0812 in the EAE model. These pharmacodynamic markers provide tools that could be applied to clinical trials to monitor CCR2 receptor engagement in future studies.

Consistent with the potent inhibition of Ly6C^hi ^monocyte trafficking in vitro and in acute in vivo inflammatory settings, we observed a dramatic reduction in Ly6C^hi ^monocytes in CNS tissues from MOG_35-55 _immunized mice treated with MK0812. The inhibition of monocyte recruitment in this disease setting would likely have the direct effect of limiting the accumulation of differentiated macrophage lineage cells capable of proinflammatory mediator production as well as secondary effects on recruitment of other cell lineages, including T lymphocytes and neutrophils, all of which were found in reduced numbers in the CNS of mice treated with MK0812. It should be noted that the impact of CCR2 antagonism by MK0812 in the EAE model may not be entirely due to inhibition of monocyte trafficking. Inhibition of other CCR2-dependent processes (e.g. through effects on the subset of T lymphocytes that express CCR2) may contribute to the efficacy that was observed in this disease setting. The relative contribution of various CCR2-expressing cell populations to the EAE disease process thus remains an important unresolved question. Irrespective of the contribution of monocytes to the effect of MK0812 in this model, our data support the utility of monitoring monocyte numbers and CCR2 ligands in the peripheral blood as pharmacodynamic measures of CCR2 antagonism *in vivo*.

## Conclusion

Here we demonstrated that CCR2 antagonism *in vivo *results in a reduction in the circulating Ly6C^hi ^monocyte population and an increase in circulating levels of the CCR2 ligands CCL2 and CCL7. We further demonstrated that CXCR4 plays a major role in the alteration in circulating monocytes under these conditions, revealing a role for CXCR4 and CCR2 in regulating monocyte homeostasis. Finally, the pharmacodynamic changes resulting from CCR2 antagonism in acute settings translated to situations of chronic drug treatment and provide potential biomarkers applicable to clinical trials with CCR2 antagonists in human populations.

## Abbreviations

AUC: area under the curve; CCL2: monocyte chemotactic protein-1; CCL3: macrophage inflammatory protein-1α; CCL7: monocyte chemotactic protein-3; CXCL12: stromal cell-derived factor-1; CNS: central nervous system; EAE: experimental autoimmune encephalomyelitis; MC: 0.4% hydroxypropyl methylcellulose; p.o.: oral gavage.

## Competing interests

The authors declare that they have no competing interests.

## Authors' contributions

YW and SHM carried out the analyses of monocyte levels and immunoassays. WG carried out analyses of MK0812 activity *in vitro*. GA, SR and JK synthesized MK0812. DL and JSF contributed to the conception and design of the studies. EPG contributed to the conception, design and interpretation of the studies and wrote the manuscript. All authors read and approved the final manuscript.
